# Hierarchical Organization of Bilateral Prefrontal‐Basal Ganglia Circuits for Response Inhibition Control

**DOI:** 10.1002/hbm.70235

**Published:** 2025-05-27

**Authors:** Liyue Lin, Yishu Chen, Zhengyuan Fan, Wei Xiong, Xuan Wang, Hongfei Ji, Jie Li, Jie Zhuang

**Affiliations:** ^1^ School of Psychology, Research Center for Exercise and Brain Science Shanghai University of Sport Shanghai China; ^2^ Translational Research Center, Shanghai Yangzhi Rehabilitation Hospital (Shanghai Sunshine Rehabilitation Center), School of Computer Science and Technology Tongji University Shanghai China

**Keywords:** concreteness effect, frontal‐basal ganglia circuits, go/no‐go lexical decision task, inhibitory demand, response inhibition

## Abstract

Response inhibition control is primarily supported by the right inferior frontal gyrus (IFG) and the prefrontal‐basal ganglia network, though the mechanisms behind right lateralization and regional interplay remain unclear. In this fMRI study, we explore the neural substrates supporting efficient inhibition control and examine whether the typical right lateralization of IFG activation can be modulated by stimulus properties (semantic features) and inhibitory demand (reaction times, RT). We chose a Go/No‐Go lexical decision task, utilizing concrete and abstract words as Go stimuli and pseudo‐word as No‐Go stimuli. Behavioral results reveal that inhibition is more effective during concrete word sessions compared to abstract word sessions, suggesting a modulation of cognitive inhibition by semantic features. Neuroimaging results further demonstrate that successful inhibition activates bilateral IFG, indicating a flexible right lateralization pattern of IFG activation that varies with stimulus properties. To examine how varying inhibitory demands modulate neural activation patterns, we reclassified concrete and abstract sessions into fast and slow sessions based on RT, followed by within‐group comparisons. Our study highlights the crucial role of the bilateral subthalamic nucleus (STN) in efficient inhibition, with increased activation associated with rapid response inhibition. Furthermore, we report enhanced neural coupling between the right IFG and multiple functionally connected regions, including bilateral insula, putamen, and pallidum, as well as between the right middle frontal gyrus and other prefrontal regions during rapid inhibitory responses, whereas no engagement of the left IFG was observed in efficient inhibition. These findings imply a hierarchical functional organization of the bilateral fronto‐basal ganglia circuits, in which the right prefrontal regions play a dominant role in inhibition control, supported by basal ganglia regions, while the left IFG may serve a supplementary function. Stimulus properties can modulate right lateralization, underscoring the dynamic and flexible nature of the prefrontal‐basal ganglia network in inhibition control.

## Graphical abstract

The functional asymmetry of the bilateral inhibitory control system in lexical decision task.

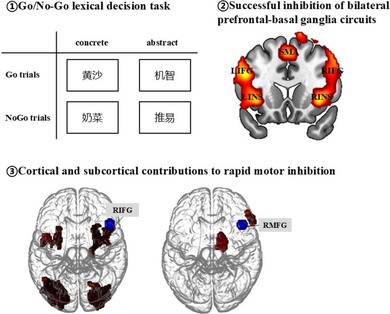

**Summary**

- Stimulus properties can modulate right lateralization of response inhibition.
- Bilateral frontal‐basal network connectivity was enhanced in rapid inhibition.
- Hierarchical organizational framework of response inhibition, dominated by the RIFG and supplemented by the LIFG.

## 1 Introduction

Response inhibition refers to the ability to suppress dominant, planned, or ongoing responses by adjusting attention, behavior, thoughts, and emotions in the face of external interference (Aron 2011). The frontal‐basal ganglia model is the most influential framework to date (Aron 2011; Aron et al. 2016). This model emphasizes the critical role of the right inferior frontal gyrus (RIFG) and the pre‐supplementary motor area (pre‐SMA) in generating the stop command. This command is subsequently relayed to the subthalamic nucleus (STN) and caudate nucleus through hyperdirect or indirect pathways, ultimately suppressing motor output via the pallidum and thalamus, thereby inhibiting responses in the primary motor cortex (M1). A notable limitation of this model is its predominant focus on right‐hemisphere networks, largely overlooking the potential contributions of the left hemisphere to inhibitory processes. However, emerging evidence challenges this right‐lateralized perspective. For instance, studies of patients with brain injuries have demonstrated that damage to the left inferior frontal gyrus (LIFG) can impair inhibitory control (Krämer et al. 2013; Swick et al. 2008). Conversely, other studies have shown that the left hemisphere can compensate for inhibitory control deficits even when the RIFG is damaged (Choo et al. 2022; Gavazzi et al. 2019), underscoring the functional significance of left‐hemisphere regions. Additionally, neuroimaging studies in healthy individuals have identified bilateral activation of the prefrontal cortex and other regions during inhibitory tasks (Baumeister et al. 2014; Leunissen et al. 2016; Tabu et al. 2012). Collectively, these findings support the development of a bilateral inhibitory control network theory, suggesting that inhibitory control may depend on distributed bilateral networks rather than exclusive right‐hemisphere specialization.

Several explanations have been proposed to account for left‐sided involvement in inhibitory network activity. First, differences in task design must be considered. The Go/No‐Go and stop‐signal tasks are the most commonly used paradigms to explore response inhibition (Hannah and Aron 2021; Schall and Godlove 2012). However, it should be noted that these two tasks explore different aspects of response inhibition, that is, response inhibition and action cancellation (Guo et al. 2018). These differences lead to variations in the neural networks involved. For instance, Go/No‐Go tasks are more likely to elicit activation in the LIFG and parietal cortex, whereas stop‐signal tasks tend to engage the RIFG more prominently (Dalley et al. 2011; Rubia et al. 2001). This may explain the right‐lateralization bias in the frontal‐basal ganglia model, as Aron's foundational studies predominantly employed the stop‐signal task (Aron 2011; Cai et al. 2012). A second explanation relates to task complexity. As research on response inhibition has advanced, task stimuli have evolved from simple shapes and letters to complex, meaningful images. For example, tasks requiring detailed scrutiny of image differences to determine inhibition demands recruit a broad network of bilateral regions, including the ventrolateral prefrontal cortex (VLPFC), anterior insula (INS), dorsolateral prefrontal cortex (DLPFC), and pre‐SMA (Köhler et al. 2018). Similarly, bilateral IFG activation has been observed in tasks using facial stimuli (Chiu and Egner 2015).

A third possibility involves the efficiency of response inhibition. Reaction times (RT) serve as an indirect measure of inhibitory demand, with demonstrated effects on inhibitory network activation patterns (Albert et al. 2013; Kok et al. 2004). Behaviorally, slower RT predicts higher inhibition success rates and greater inhibitory demand, whereas faster responses increase the probability of inhibition failures, reflecting reduced inhibitory demands due to automatic response tendencies. Hirose et al. (2012) established key neural efficiency markers in Go/No‐Go tasks, identifying positive correlations between inhibition efficiency and activation intensity in left‐hemisphere regions including the inferior frontal gyrus, superior frontal gyrus (SFG), precentral/middle frontal gyrus (PreCG/MFG), and temporoparietal junction (TPJ). This left‐lateralized activation pattern suggests that under conditions of high inhibitory demand, the brain may recruit complementary left‐hemisphere mechanisms while maintaining core right‐hemisphere inhibitory functions. Notably, these findings challenge the conventional right‐hemisphere dominance paradigm (Gavazzi et al. 2023), but are consistent with the idea that task complexity affects lateralization, that high inhibitory needs may induce compensatory activation in atypical hemispheres, such as the left hemisphere. This interpretation is supported by data from the Slow‐RT group (M = 548 ms, SD = 123 ms), where slower responses—characteristic of complex task conditions—were associated with RIFG engagement alongside bilateral recruitment of prefrontal, parietal, and insula regions. This activation pattern implies that prolonged reaction times may reflect strategic cognitive compensation (e.g., working memory allocation, attentional modulation) that reduces reliance on unilateral networks in favor of bilateral resource mobilization for complex control processes (Zhang and Li 2012). Collectively, these findings suggest that inhibitory function may be subserved by a complementary system: while the right hemisphere maintains general inhibitory capacity, the left hemisphere provides efficiency optimization, with their relative contributions dynamically modulated by task demands and behavioral objectives.

Research on the discovery and interpretation of bilateral inhibitory networks has made gradual progress, yet many questions remain unresolved. Overall, the right‐lateralized inhibitory network appears dominant, with the RIFC, pre‐SMA, and insula being the most consistently activated regions. However, detecting activity in the STN remains challenging. Additionally, the left inhibitory network exhibits considerable variability, and no studies have systematically identified tasks that reliably engage this network, which limits the development of a comprehensive bilateral inhibition model. To address these gaps and refine the response inhibition model, we aimed to design a task capable of stably activating bilateral inhibitory networks. Building upon the left‐lateralized inhibitory network framework, we employed Chinese compound words as stimuli in a Go/No‐Go task, which is often used to examine semantic processing during word recognition (Danguecan and Buchanan 2016; Gomez et al. 2007; Lee et al. 2019; Vergara‐Martínez et al. 2020). While previous research using this paradigm has primarily focused on semantic effects during Go processing, no studies to date have specifically investigated response inhibition mechanisms during No‐Go processing. We therefore systematically manipulated semantic features to test the hypothesis that the right‐lateralized activation pattern of the IFG can be modulated by linguistic properties and to explore its interactions with other brain regions. Given the left‐lateralized nature of language processing (Ji et al. 2019), this approach may better engage left‐hemisphere networks. To ensure semantic processing of the stimuli, we incorporated the lexical semantic effect of concreteness (Danguecan and Buchanan 2016), distinguishing between concrete words, which evoke sensory experiences, and abstract words, which rely on conceptual representations (Dove 2016). Prior research has demonstrated that abstract words elicit stronger activation in the LIFG compared to concrete words (Binder et al. 2009; Della Rosa et al. 2018; Hoffman et al. 2015), making this region particularly relevant to our study. RT during Go trials was used as an index of inhibitory demand, allowing us to explore brain regions involved in rapid response inhibition, with a specific focus on the role of the left‐lateralized network. This approach provides novel insights into the neurocognitive basis of inhibitory control and elucidates the critical contribution of left‐hemispheric systems to efficient behavioral inhibition.

## 2 Materials and Methods

### 2.1 Participants

Twenty healthy adult volunteers (10 males, mean age: 21.55 ± 1.23 years) without any history of neurological or psychiatric disorders participated in the present fMRI study. All participants had normal or corrected‐to‐normal vision, and the Edinburgh Handedness Inventory was used to determine that all participants were right‐handed (Oldfield 1971; Veale 2014). Informed consent was obtained from each participant prior to the study, in accordance with the Declaration of Helsinki. The study was approved by the Shanghai University of Sports (registration number 102772022RT079), and participants were compensated for their time.

### 2.2 Experimental Design

E‐prime software (version 2.0, Neurobehavioral Systems Inc.) was used to present the stimuli and record the responses. Visual stimuli were displayed using an LCD projector, which projected the images onto a screen positioned behind the participants' heads. The participants viewed the screen through an angled mirror attached to the head coil of the MRI setup. Prior to the fMRI scan, each participant was instructed to read and complete a brief practice task to familiarize themselves with the procedure.

A Go/No‐Go lexical decision task was employed (Danguecan and Buchanan 2016). During the task, two characters were presented on the screen (font size: 200 pt). Participants were instructed to press the right index finger as quickly and accurately as possible when they saw a real word (Go trial). If the word was a pseudo‐word (NoGo trial), no key press was required. The inter‐trial interval (ITI) between two consecutive Go trials was 1000, 1100, 1200, 1300, or 1400 ms, and the ITI for each Nogo trial and the subsequent Go trial was 3700, 4300, 4900, 5500, 6100, or 6700 ms, presented in a pseudo‐random manner, with an equal probability of occurrence (20% and 16.7%, respectively) for each value. The experiment consisted of four counterbalanced sessions, with two sessions employing concrete words as real words and the other two utilizing abstract words. To control for potential order effects, participants were randomly assigned to one of two presentation sequences: (1) two concrete word sessions followed by two abstract word sessions, or (2) the reverse order. Each session consisted of 120 Go trials and 30 NoGo trials.

A total of 240 abstract nouns and 240 concrete nouns were selected as stimuli from the 1986 Chinese Frequency Dictionary. All words were two‐character Chinese compound words, with the frequency of both noun categories ranging from 100 to 500 occurrences per million words. Additionally, two non‐combinable single words from the thesaurus were selected as pseudo‐words, for a total of 120.

### 2.3 Data Acquisition and Analysis

#### 2.3.1 Statistical Analysis of Behavior

For each participant, RTs and accuracy were calculated for Go trials, and false alarms for NoGo trials, in both the concrete and abstract sessions. Trials with RTs less than 100 ms or greater than 3 standard deviations were excluded, resulting in the removal of 1.18% of the total trials. To compare recognition performance between the two word categories, R software was used to perform paired *t*‐tests for both RTs, accuracy, and false alarms.

#### 2.3.2 Regrouping of Subjects and Sessions

To elucidate the absence of a significant difference in RTs between the two word categories, participants were divided into two groups based on their RT patterns: Group 1 exhibited longer RTs for concrete words, while Group 2 demonstrated longer RTs for abstract words. Interestingly, each group comprised an equal number of participants (*n* = 10). A two‐way mixed‐design analysis of variance (ANOVA) was performed with group (Group 1 vs. Group 2) as a between‐subjects factor and word type (concrete vs. abstract) as a within‐subjects factor. The data of all 20 participants were exclusively used for behavioral analysis to assess whether there are individual differences in concreteness effects.

In our neuroimaging analysis, we examined how varying inhibitory demands modulate the response inhibition network while controlling for lexical features. Rather than grouping across participants, we implemented a within‐subject design by reclassifying sessions based on RT patterns. For each participant, we first established baseline RT measures by calculating mean RTs separately for concrete and abstract word sessions. Sessions were then categorized as fast or slow sessions relative to these individual baseline measures: concrete word sessions were classified as slow sessions when their mean RT exceeded that of abstract, and vice versa. This approach allowed us to compare fast versus slow inhibition conditions while maintaining within‐subject consistency.

#### 2.3.3 Imaging Data Acquisition and Statistical Analysis

All participants were scanned using a 3.0 Tesla GEMR 750 whole‐body MRI scanner (General Electric, Milwaukee, Wisconsin, USA) with an eight‐channel head coil at Tongji University. Functional images were acquired using a gradient‐echo EPI sequence with the following parameters: TR = 2 s, TE = 23 ms, flip angle = 77°, voxel size = 3 × 3 × 3 mm, FOV = 19.2 × 19.2 cm, and 40 slices (no gap). T1‐weighted structural images were also collected using a 3D fSPGR pulse sequence for anatomical localization, with the following parameters: 162 contiguous slices, TR = 7.64 s, TE = 2.94 ms, flip angle = 12°, voxel size = 1 × 1 × 1 mm, and FOV = 25.6 cm^2^.

Preprocessing and statistical analysis of the functional and structural images were performed in SPM12 (Wellcome Institute of Cognitive Neurology, London, UK, http://www.fil.ion.ucl.ac.uk) under MATLAB (MathWorks Inc., Natick, MA, USA). First, slice timing correction was applied to correct for acquisition time differences. Head motion realignment was then performed, and participants with head translations greater than 3 mm or rotations exceeding 3° were excluded. The head motion of all remaining images was within acceptable limits. The corrected images were spatially normalized to the Montreal Neurological Institute (MNI) space using the EPI template provided by SPM, with voxel resampling to 3 mm × 3 mm × 3 mm. Gaussian smoothing with a full‐width at half‐maximum (FWHM) of 8 mm was applied to reduce spatial noise.

For first‐level analyses, a general linear model (GLM) was used to construct a multiple regression design matrix that included five event types: AbstractGo, AbstractNoGo, ConcreteGo, ConcreteNoGo, and error. These events were time‐locked to the onset of each trial using a canonical synthetic hemodynamic response function (HRF) and its first‐order time derivative, with an event duration of 0 s. The six realignment parameters (translations and rotations along the three spatial axes) were included as covariates to account for residual head movement artifacts. Trials involving errors were treated as a nuisance variable, and only correct Go and NoGo trials were analyzed. For second‐level analyses, two within‐subject variables, word (Abstract/Concrete) and condition (Go/NoGo), were modeled. An *F*‐test was conducted to examine the main effects and interactions between these two variables.

After regrouping sessions according to the RT, first‐ and second‐level analyses were performed again. First‐level analyses were then performed using a GLM that included the following event types: FastGo, FastNoGo, SlowGo, SlowNoGo, error, and the six motion parameters. A canonical HRF was used to model each trial, including its first‐order time derivative, with an event duration of 0 s. Error trials included both incorrect Go and NoGo trials. Contrasts for FastNoGo > FastGo and SlowNoGo > SlowGo were computed for each subject and used in second‐level analyses. For second‐level analyses, one between‐subject variable (Group: 1/2) and one within‐subject variable (speed: Fast/Slow) were modeled, and an *F*‐test was used to examine the main effects and interactions of these variables.

Significant activations were reported at *p* < 0.001 or *p* < 0.005 (uncorrected at the voxel level), and *p* < 0.05 (corrected at the cluster level using the false discovery rate). All peak coordinates of significant clusters were reported in MNI space. The STN template was derived from a recent study using ultrahigh 7T scans (Keuken et al. 2014).

#### 2.3.4 Generalized Psychophysiological Interaction (gPPI) Analysis

To examine the functional connectivity modulated by task conditions, we conducted a generalized psychophysiological interaction (gPPI) analysis. This whole‐brain seed‐to‐voxel analysis was performed using the CONN toolbox (https://web.conn‐toolbox.org/). Based on the activation results from the contrast NoGo > Go, the activation peak coordinates of eight regions of interest (ROIs) were defined as the centers for 8‐mm radius circular ROIs. The specific coordinates for these ROIs were as follows: LIFG (*x* = −46 *y* = 22 *z* = 16), RIFG (*x* = 46 *y* = 21 *z* = 20), LMFG (*x* = −48 *y* = 17 *z* = 40), RMFG (*x* = 43 *y* = 20 *z* = 40), LSMA (*x* = −3 *y* = 17 *z* = 53), RSMA (*x* = 3 *y* = 17 *z* = 53), LINS (*x* = −35 *y* = 19 *z* = 2), and RINS (*x* = 37 *y* = 21 *z* = 0). The deconvolved time series from these ROIs were extracted to create the physiological variable. The PPI‐GLM included the physiological variable (time series of the ROI), the psychological variable (NoGo > Go and FastNoGo > SlowNoGo), the PPI interaction term, and the motion parameters. The PPI interaction terms were created by combining the physiological and psychological terms. The significance threshold was set at the voxel level (*p* < 0.005, uncorrected) and at the cluster level (*p* < 0.05, false discovery rate corrected).

## 3 Results

### 3.1 Behavioral Results

**FIGURE 1:**
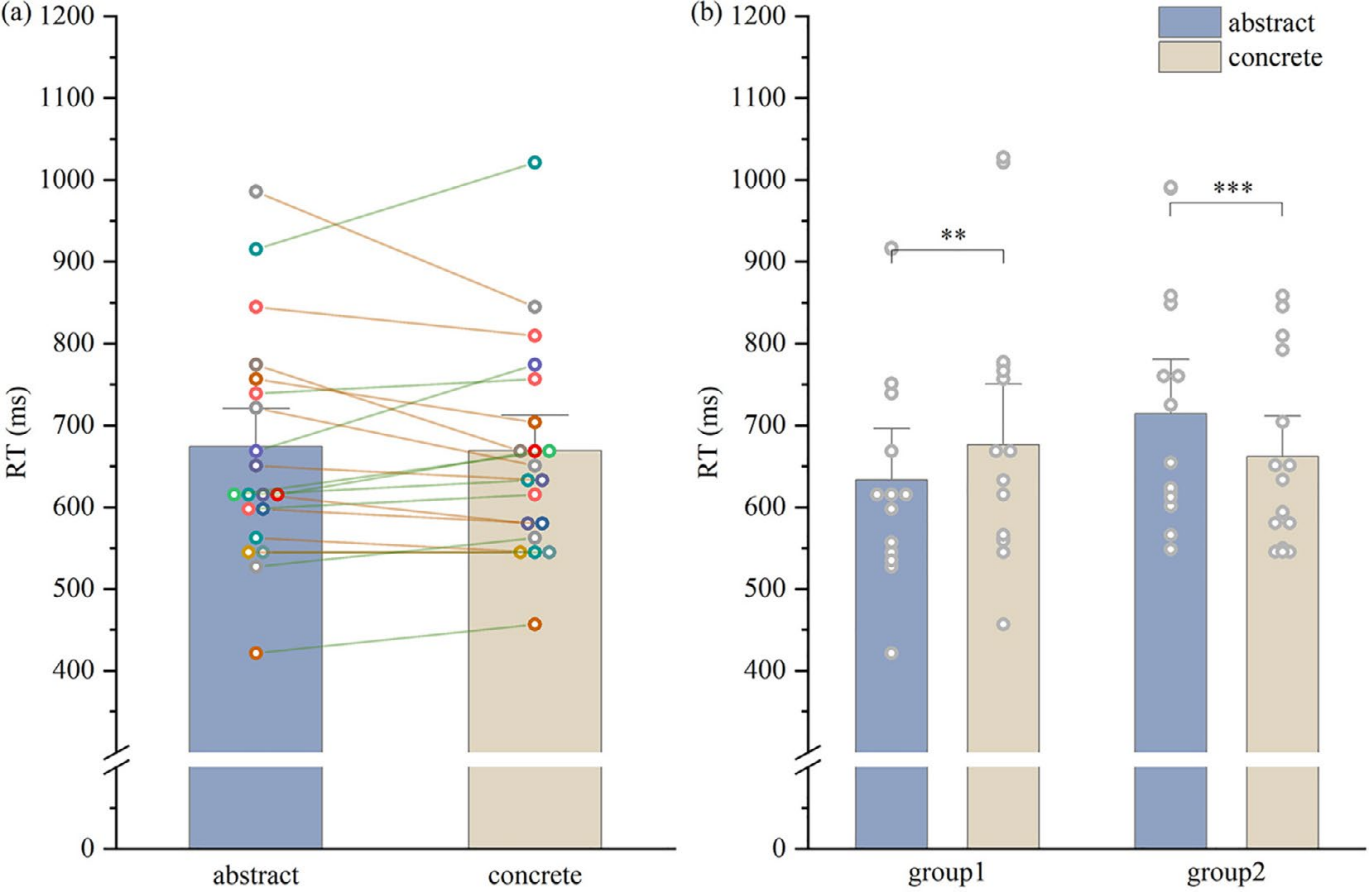
RTs for Go trials with abstract and concrete words. (a) Average RTs across all subjects, with individual subject data presented. (b) Subjects were grouped based on their RTs for abstract and concrete words. Group 1 showed shorter RTs for abstract words compared to concrete words, while Group 2 exhibited the opposite pattern. **p < 0.01, ***p < 0.001.

False alarms, RTs, and accuracy data were analyzed using a paired *t*‐test to assess performance differences between abstract and concrete words. A significant difference in false alarms was found between concrete and abstract NoGo trials (concrete = 7.50% ± 6.39%, abstract = 12.42 ± 11.64%; *t* = 2.970; *p* = 0.008). However, no significant differences were observed in either RTs (Figure 1a, concrete = 668.98 ± 129.73 ms, abstract = 673.93 ± 138.74 ms; *t* = −0.353; *p* = 0.728) or accuracy (concrete = 97.88% ± 1.21%, abstract = 97.65% ± 2.54%; *t* = 0.463; *p* = 0.649) between concrete and abstract Go trials (see Figure 1a).

A group × word ANOVA was conducted on the RTs. Neither the main effects of group (*F* = 0.311, *p* = 0.584, *η*_*p*_^*2*^ = 0.017) nor word (*F* = 0.298, *p* = 0.592, *η*_*p*_^*2*^ = 0.016) were significant, but the interaction effect was significant (*F* = 27.567, *p* < 0.001, *η*_*p*_^*2*^ = 0.605) (Figure 1b). In Group 1, RTs for concrete words were significantly greater than for abstract words (*p* = 0.004), whereas in Group 2, RTs for abstract words were significantly greater than for concrete words (*p* < 0.001, Figure 1b). These results suggest that the lack of an overall difference in RTs may be attributed to individual differences among participants.

### 3.2 Imaging Results

#### 3.2.1 Cortical and Subcortical Activation During Inhibition

**FIGURE 2:**
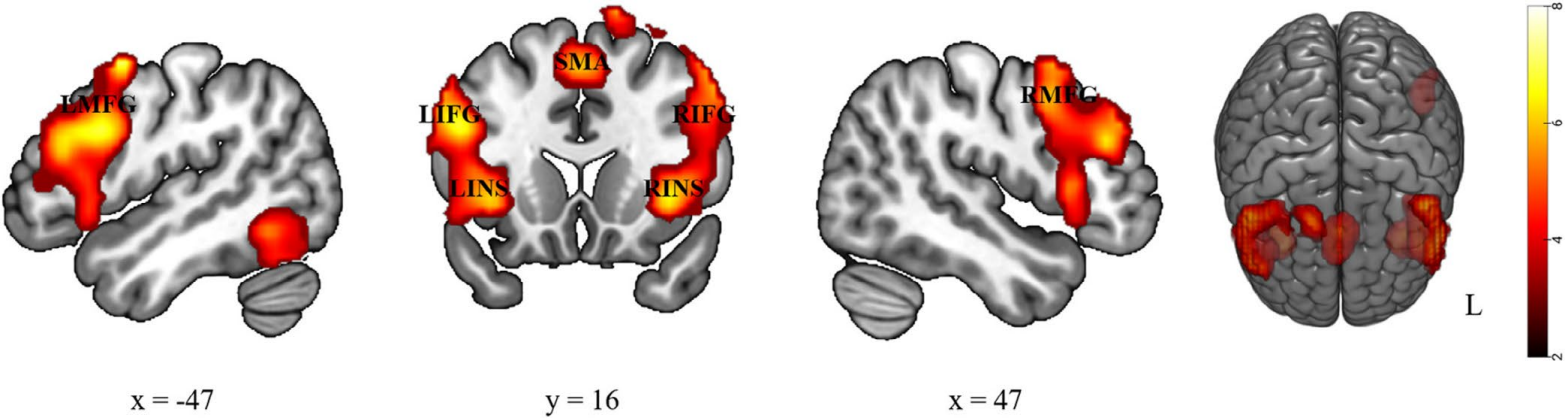
Activation during NoGo > Go for all subjects (*N* = 20). Correction thresholds were voxel‐level uncorrected (*p* < 0.001) and cluster‐level FDR corrected (*p* < 0.05).

To investigate potential differences in the recognition of concrete and abstract words, we compared the correct Go trials for both word types. However, no significant activation differences were observed. To further examine the brain regions associated with successful inhibition during the Go/No‐Go lexical decision task, we conducted contrasts for NoGo > Go. This analysis revealed significant activation in the bilateral IFG, MFG, SFG, SMA, and INS (Table 1a, Figure 2). These regions are classically associated with inhibitory control. Interestingly, we observed more activation in the left hemisphere.

**TABLE 1:** Brain regions showing significant relative increases in the bold response associated with NoGo > Go and Fast > Slow.

Area	Cluster size (mm^3^)	Peak MNI (mm)	*t*
**a. Nogo** > **Go**			
		33 26 5	8.28
RIFG	408		
RSMA	86		
LSMA	91		
RMFG	280		
RSFG	92		
LSFG	54		
RINS	115		
RPrecentral	73		
RMCC	16		
		−54 23 32	7.92
LIFG	540		
LMFG	83		
LINS	116		
LPrecentral	171		
		−48 −55 −13	5.39
LITG	74		
LIOG	35		
LFFG	21		
LMTG	13		
LCerebellar Crus	13		
**b. Fast** > **Slow**			
		−12 −10 −13	4.52
LSTN	8		
LTHAL	94		
RSTN	15	12 –7 −10	4.35
		−30 −52 44	4.34
LParietal	243		
		30 –40 53	4.06
RPostcentral	130		
RParietal	80		
RPrecentral	30		

*Note:* The coordinates (x, y, z) correspond to MNI coordinates. Correction thresholds were voxel‐level uncorrected (NoGo > Go: *p* < 0.001, Fast > Slow: *p* < 0.005) and cluster‐level FDR corrected (*p* < 0.05).

Abbreviations: FFG = fusiform gyrus, IFG = inferior frontal gyrus, INS = insula, IOG = inferior occipital gyrus, ITG = inferior temporal gyrus, MCC = middle cingulate cortex, MFG = middle frontal gyrus, MTG = middle temporal gyrus, SFG = superior frontal gyrus, SMA = supplementary motor area, STN = subthalamic nucleus, THAL = thalamus.

**FIGURE 3:**
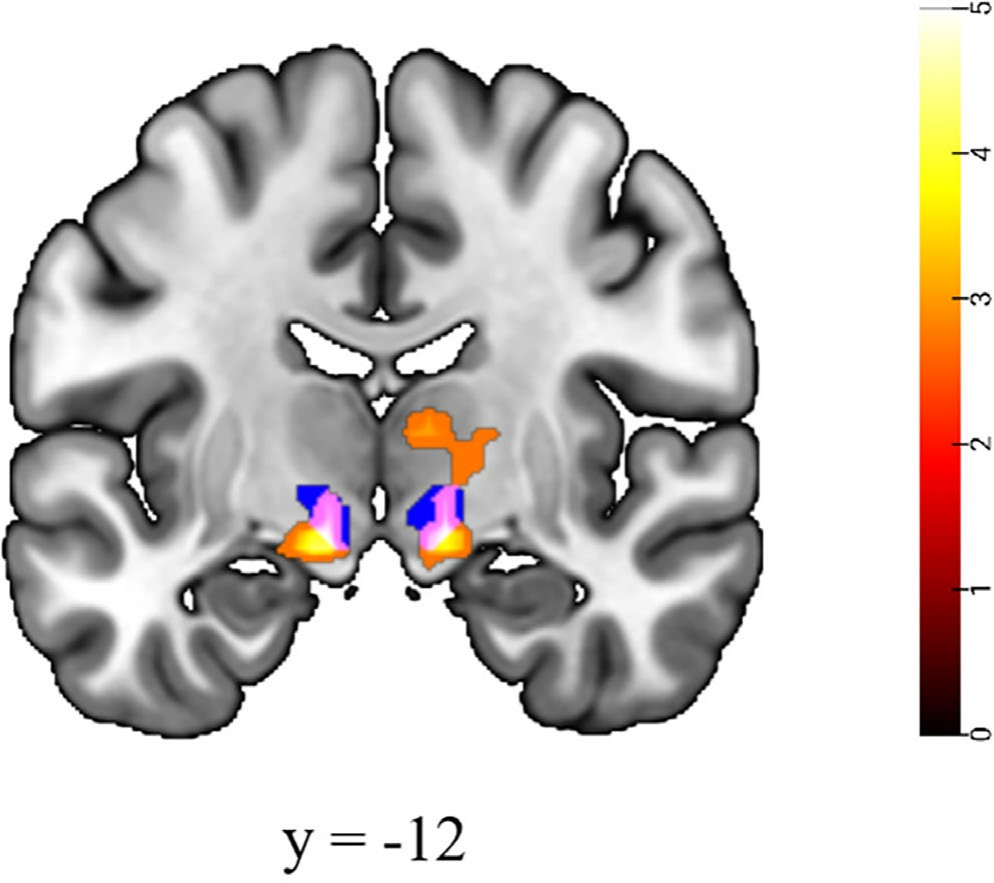
Activation during (FastNoGo–FastGo) > (SlowNoGo–SlowGo) for all subjects (*N* = 20). The blue area is the bilateral STN template, the hot area is the Fast > Slow activation, and the purple area is the STN activated by Fast > Slow. Correction thresholds were voxel‐level uncorrected (*p* < 0.005) and cluster‐level FDR corrected (*p* < 0.05).

When grouping subjects based on behavioral data, no group differences were found in the NoGo > Go contrast. However, when contrasting fast versus slow NoGo > Go trials, we identified bilateral STN activation in the fast > slow condition, but no significant activation was observed in the slow > fast condition (Table 1b, Figure 3).

#### 3.2.2 General Psychophysiological Interaction (gPPI) Analysis With the RIFG and RMFG as the Source Region

**FIGURE 4:**
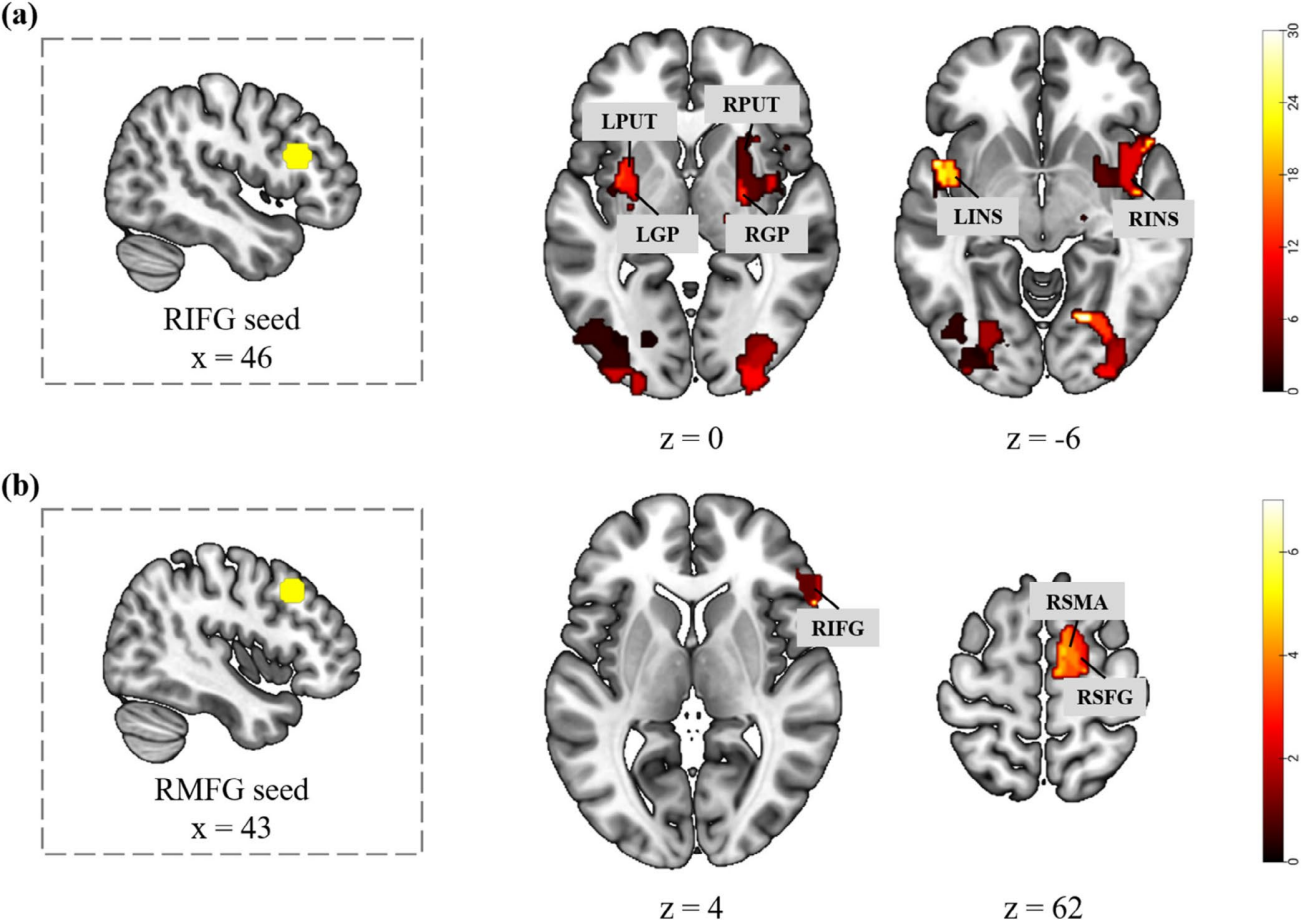
Results of the gPPI analysis, with the RIFG (a) and RMFG (b) as the seed regions and the differential processing of the task types (i.e., FastNoGo > SlowNoGo) as the psychological factor. Correction thresholds were voxel‐level uncorrected (*p* < 0.005) and cluster‐level FDR corrected (*p* < 0.05). GP = Pallidum, PUT = Putamen.

Successful inhibition relies on the synergistic cooperation of multiple brain regions. In this study, we identified eight regions that were significantly activated in the NoGo > Go contrast and are strongly associated with response inhibition. These regions were used as seed points to examine their functional connectivity with other brain regions during the NoGo > Go and FastNoGo > SlowNoGo contrasts. For NoGo > Go, no significant results were observed. However, functional connectivity between the RIFG and RMFG with other brain regions was observed during FastNoGo > SlowNoGo, but not with the other six seed points. Specifically, the RIFG exhibited increased neural coupling with the bilateral insula, putamen, and pallidum (Figure 4a), while the RMFG showed increased neural coupling with the right IFG, SMA, and SFG (Figure 4b).

## 4 Discussion

The present study investigated the activation of inhibitory networks during a Go/No‐Go task using word stimuli, examining how concreteness effect and inhibitory demand influence this activation. The key findings are as follows: (1) Successful inhibition during the lexical decision task engaged bilateral inhibitory control networks, including the bilateral IFG, MFG, SFG, SMA, and INS. Notably, left‐hemisphere activation was stronger and more extensive than reported in previous studies. (2) The specificity effect was observed only during pseudoword recognition, indicating that semantic features modulate inhibitory control to some extent. (3) Rapid response inhibition was associated with increased bilateral STN activation, underscoring the STN's critical role in efficient inhibition. (4) Functional connectivity analyses revealed increased neural coupling between the RIFG and the bilateral INS, putamen, and pallidum during rapid inhibition, while the RMFG showed enhanced coupling with the right IFG, SMA, and SFG. These findings emphasize the importance of bilateral network dynamics and suggest that current models of inhibitory control should be expanded to account for both the right hemisphere's dominance in adaptive cognitive control and the synergistic interactions between hemispheres.

### 4.1 The Concreteness Effect: Individual and Task Differences

Concrete and abstract words engage different processing mechanisms due to their varying levels of perceptibility, a phenomenon known as the concreteness or imageability effect (Kearney et al. 2025; Yan et al. 2023). Typically, concrete words are processed more efficiently than abstract words across various tasks (Chen and Lin 2012; Pexman et al. 2017; Tse and Altarriba 2022; Zdrazilova and Pexman 2013). Neuroimaging studies have shown that abstract words produce greater activation in the right frontal and left temporal regions, whereas concrete words engage the left occipital and parietal lobes (Acheson and Hagoort 2013; Hoffman et al. 2015; Kosslyn et al. 2001).

In contrast to these findings, the present study did not observe significant differences in RTs, accuracy, and activation patterns between concrete and abstract words in Go trials. The lack of accuracy differences may result from a ceiling effect, as participants achieved near‐perfect recognition (up to 97% accuracy), consistent with previous research (Tsai et al. 2009; Xiao et al. 2012). The absence of RT differences across all subjects is likely to reflect individual variability. A concreteness effect did manifest among participants, although only for half of them. Another reason may be that the Go/No‐Go task used in this study differs from lexical decision tasks that emphasize explicit recall and recognition (Xiao et al. 2012). The Go/No‐Go paradigm requires fewer cognitive resources and provokes faster responses (Lee et al. 2019). These features are not conducive to facilitating deep semantic processing and thus weaken the specific effects observed (Dufau et al. 2015; Tsai et al. 2009), as suggested by the theories of contextual validity (Schwanenflugel et al. 1992) and double coding (Paivio et al. 2000).

Although no concreteness effect was observed for real words, the processing of real words in this study appeared to influence subsequent pseudoword recognition. Specifically, false alarm rates were significantly lower for concrete than abstract sessions. Given that pseudowords were matched for linguistic properties (e.g., specificity) across sessions, this difference likely stems entirely from the differential processing of real words. That is, the recognition of concrete versus abstract words differentially affected subsequent pseudoword processing. We propose that the established concreteness effect (wherein concrete words require fewer cognitive resources than abstract words) allowed participants to allocate more residual resources to successful inhibition when pseudowords appeared. However, comparing these findings with previous research is challenging because of the unique design of our Go/No‐Go task and the limited research on semantic neighborhood effects in this paradigm (Danguecan and Buchanan 2016).

### 4.2 Bilateral Inhibitory Networks Activated in Go/No‐Go Lexical Decision Task

As demonstrated in prior research, fMRI studies have shown that specific brain regions are activated in response to the presentation of letters (Fryer et al. 2019; Happer et al. 2021; Lesage et al. 2020; Menon et al. 2001), images (Brown et al. 2015; He et al. 2019; Xiao et al. 2021), and shapes (Pan and Wang 2023). This activation is often bilateral, indicating that both hemispheres contribute to processing such stimuli. Similar findings of bilateral network activation have been observed in studies of response inhibition. Our results align with these observations, demonstrating that response inhibition engages the IFG, SMA, and basal ganglia, with significant bilateral involvement. These findings suggest that the activation of the left inhibitory network may be modulated by task complexity, with more complex tasks requiring greater cognitive control. The left frontal lobe, particularly the IFG, is highly specialized in enforcing task rules and updating cognitive processes (Moutsiana et al. 2015; Zhu et al. 2020). Additionally, the activation of bilateral MFG and SFG may reflect the need for additional cognitive resources to manage competing demands and maintain task goals (Apšvalka et al. 2022; Depue et al. 2016).

The primary finding of this study is reflected in the observation of comparable activation between right and left hemispheric inhibitory control networks. While prior research has consistently demonstrated right‐lateralized dominance in inhibitory control (Diesburg and Wessel 2021; Jahanshahi et al. 2015), our findings challenge the conventional explanation that left‐hemispheric activation solely reflects task complexity. Recent meta‐analytic evidence from Go/No‐Go studies indicates that complex tasks (characterized by heightened working memory demands or stimulus complexity) elicit increased left‐hemispheric activation, especially left INS, but this pattern generally excludes the LIFG (367 mm^3^) (Aziz‐Safaie et al. 2024). Our findings demonstrate more extensive left‐hemisphere engagement, with larger activation clusters encompassing the left IFG (540 mm^3^), MFG (83 mm^3^), SFG (54 mm^3^), SMA (91 mm^3^), and INS (116 mm^3^). This broader activation pattern suggests that our task may recruit additional cognitive resources beyond those typically observed in standard inhibition tasks. The present study found large activations in the LIFG, which may be attributable to the initial hypothesis that activation of bilateral brain regions may be due to the use of words as stimuli (Happer et al. 2021; Jimura et al. 2009; Lesage et al. 2020). As the Go and Nogo trials were both Chinese words with linguistic properties balanced between them, the contrast of Nogo minus Go trials cannot generate an effect of linguistic properties. Therefore, the LIFG activation in our response inhibition effect is not a language processing effect; rather, it reflects the modulation of linguistic properties of stimuli in the process of response inhibition.

This study lends support to the hypothesis that as task complexity increases, additional brain regions are recruited to meet the inhibitory demands, as evidenced by the greater involvement of the bilateral INS, thus highlighting the adaptive nature of the inhibitory network (Aron et al. 2016). Furthermore, stimulus modality appears to exert significant top‐down modulation on regional involvement within the inhibitory control network. This is particularly evident in our observation of LIFG activation during lexical inhibitory tasks, suggesting domain‐specific neural specialization within the broader inhibitory network.

### 4.3 Cortical and Subcortical Contributions to Rapid Motor Inhibition

Inhibitory control is closely linked to attentional processes, often assessed through RTs in Go trials (Kelly et al. 2008; MacDonald et al. 2006; Thompson et al. 2021). Longer RTs in Go trials are generally associated with a higher likelihood of successful inhibition and a reduced need for activation in the frontal–parietal regions (Albert et al. 2013; Chikazoe et al. 2009).

First, we sought to identify key regions underlying rapid response inhibition by comparing neural activation patterns between fast and slow inhibition trials. Interestingly, bilateral STN activation was selectively observed during rapid inhibition, but not during general inhibition conditions. This finding aligns with existing theoretical frameworks suggesting that distinct frontal cortical regions may engage the STN through hyperdirect pathways to mediate global inhibition (Aron et al. 2014, 2016; Guo et al. 2018; Nambu et al. 2002). However, direct anatomical and physiological evidence for this pathway in humans remains limited, and the STN's involvement in cognitive control often requires ROI analysis for detection (Guo et al. 2018). This methodological constraint likely explains the absence of STN activation in our conventional No‐go > Go contrast. The selective emergence of STN activation in fast versus slow comparisons may reflect the ultra‐rapid (~2 ms) functional connectivity between STN and IFG (Chen et al. 2020), which provides a neural basis for rapid response inhibition. Supporting this interpretation, Cai et al. (2019) demonstrated a positive correlation between STN activation intensity and inhibition speed, suggesting greater STN recruitment during faster inhibition. Collectively, these findings underscore the critical role of STN in rapid response inhibition.

We then identified eight regions of interest based on the NoGo > Go contrast and subsequently conducted a comparative analysis of functional connectivity related to fast and slow motor inhibition using the gPPI approach. This analysis highlighted cortical and subcortical contributions to rapid motor inhibition, specifically enhanced neural coupling of the RIFG and RMFG to other critical regions. Notably, no significant connectivity was found in the NoGo > Go comparison, suggesting that functional synergies become evident only when inhibitory demands are increased.

Enhanced neural coupling between RIFG and the bilateral insula, putamen, and pallidum was observed during rapid inhibition (Guo et al. 2018; Jahfari et al. 2019). Interestingly, our results indicate that rapid response inhibition engages subcortical regions bilaterally, rather than predominantly activating the right hemisphere. This suggests that tasks requiring greater conflict monitoring and inhibition may necessitate the coordinated involvement of bilateral networks, rather than relying on unilateral control. Such bilateral coordination may be particularly relevant for tasks of increased complexity or those involving multiple inputs, such as language stimuli (Aron et al. 2016; Jahanshahi et al. 2015; Munakata et al. 2011).

Additionally, the RMFG exhibited enhanced neural coupling with cortical areas such as the RIFG, RSMA, and RSFG during rapid response inhibition. This finding suggests that the RMFG plays an auxiliary role in supporting the RIFG (Fonken et al. 2016; Swann et al. 2013) and RSMA (Hu et al. 2015; Takeyama et al. 2022) for the rapid execution of inhibition by integrating conflict detection and attentional modulation information. Furthermore, the RMFG works in conjunction with the RSFG to dynamically allocate attentional resources, focusing on task‐relevant stimuli and blocking distracting information (Friedrich and Beste 2020; Li et al. 2013). A recent meta‐analysis corroborated these findings, showing that the RMFG/SFG is activated under high inhibition demands (i.e., during fast inhibition) (Gavazzi et al. 2023). Considering these findings, we propose that the RMFG serves as a functional hub in fast response inhibition tasks, facilitating the integration and coordination of various functional modules.

### 4.4 Functional Asymmetry in a Bilateral Inhibitory Control System

Our analyses revealed a notable absence of significant functional connectivity between left prefrontal regions and other nodes of the bilateral fronto‐basal ganglia network during efficient response inhibition. This finding suggests that the left prefrontal cortex may not be directly engaged in core inhibitory processes under these experimental conditions. Collectively, these results support a hierarchical functional organization of the fronto‐basal ganglia system, characterized by: (1) right prefrontal dominance in inhibitory control, particularly the RIFG; (2) supplementary involvement of the LIFG in complementary cognitive functions. This hemispheric specialization becomes particularly pronounced under high‐demand conditions, such as during rapid response inhibition.

The current findings demonstrate that the inhibitory control network exhibits both bilateral structural engagement and demand‐dependent functional lateralization. Whole‐brain analyses (Nogo > Go) revealed robust activation throughout bilateral prefrontal cortices (inferior/middle/superior frontal gyri), insula, and SMA, confirming the distributed nature of this system. However, gPPI analyses identified a right‐lateralized connectivity pattern during fast inhibition, with the RIFG showing enhanced coupling with subcortical structures (bilateral insula, putamen and pallidum) and the RMFG exhibiting stronger integration with other right‐lateralized frontal regions (e.g., RSMA, RSFG). This functional asymmetry corroborates the well‐established right‐hemisphere advantage in reactive stopping (Aron et al. 2014), wherein rapid inhibition critically depends on efficient right prefrontal‐subcortical communication.

Importantly, this right‐lateralized pattern coexists with the network's fundamental bilateral architecture. We propose that the system dynamically reconfigures under heightened inhibitory demands: while the right hemisphere assumes primary control of urgent stopping, the left hemisphere (particularly LIFG) may transition to support functions like semantic processing, conflict monitoring, or error processing (Jahanshahi et al. 2015). This flexible specialization accounts for clinical evidence showing that right‐hemisphere lesions produce more severe stopping impairments, while left‐hemisphere contributions remain behaviorally significant in complex contexts (Swick et al. 2008). Future research should systematically investigate how left prefrontal regions contribute to distinct inhibitory demands (e.g., proactive versus reactive control) to further elucidate this bilateral‐asymmetric interplay.

### 4.5 Limitations

This study has several methodological limitations that warrant discussion. First, while response inhibition effects have been reliably demonstrated in small samples (Lesage et al. 2020; Suttkus et al. 2021; Vanova et al. 2023), our sample size of 20 participants remains relatively small. Future research would benefit from larger samples to increase statistical power and allow for subgroup analyses. Second, in line with most of the existing literature, we recruited only right‐handed participants and required right‐handed responses. Although previous research reveals no significant differences in inhibitory performance between left‐ and right‐handed individuals (Mancini and Mirabella 2021; Schrammen et al. 2020; Serrien and Sovijärvi‐Spapé 2013), no neuroimaging studies to date have conclusively demonstrated whether handedness influences inhibitory network asymmetry. We hypothesize that the observed left‐hemisphere inhibitory network activation does not solely reflect response hand effects, as previous demonstrations of right‐lateralized inhibition typically involved right‐handed participants making right‐handed responses without yielding left‐hemisphere activation patterns. Nevertheless, this remains an open question that deserves further investigation, and future studies should consider the balance in the number of subjects with different handedness when incorporating them in their study designs.

## 5 Conclusion

This study advances our understanding of the neural mechanisms underlying bilateral inhibitory control and its interplay with semantic processing and inhibitory demand. The findings reveal symmetrical activation patterns in bilateral inhibitory networks during successful inhibition in cognitively demanding tasks, particularly in lexical Go/No‐Go paradigms. Notably, rapid inhibition was associated with both the bilateral STN and distinct neural pathways mediated by the RIFG and RMFG. These results demonstrate functional hemispheric specialization, with the right frontal lobe assuming a primary role in rapid response inhibition, while the left frontal lobe serves a complementary function, establishing a hierarchical organizational framework.

Although no significant concreteness effect was observed, the study highlights the critical influence of individual variability and task parameters on inhibitory control processes. Future investigations should aim to delineate the precise lexical characteristics that modulate inhibitory control within this paradigm. Nevertheless, the current lexical decision task provides a robust framework for investigating bilateral inhibitory network activation.

In summary, detailed examination of bilateral inhibitory networks may elucidate the integrative and coordinative mechanisms of cognitive inhibition. Such research could enhance our understanding of how inhibitory networks adapt to complex cognitive demands and inform therapeutic interventions for disorders characterized by inhibitory control deficits.

## Ethics Statement

The study was conducted in accordance with the Declaration of Helsinki and approved by the Scientific Research Ethics Committee of Shanghai University of Sport (registration number 102772022RT079).

## Data Availability

The data that support the findings of this study are available from the corresponding author upon reasonable request.
